# Epstein-barr virus induced cellular changes in nasal mucosa

**DOI:** 10.1186/1743-422X-3-6

**Published:** 2006-02-01

**Authors:** Matteo Gelardi, Marilena Tomaiuolo, Michele Cassano, Gaspare Besozzi, Maria Luisa Fiorella, Agata Calvario, Maria Antonia Castellano, Pasquale Cassano

**Affiliations:** 1 Department of Otolaryngology, University of Bari, P.zza G. Cesare, 70120, Bari, Italy; 2 Virology Institute, University of Bari, P.zza G. Cesare, 70120, Bari, Italy; 3 Electron Microscope Institute, University of Bari, P.zza G. Cesare, 70120 Bari, Italy; 4 Department of Otolaryngology, Ospedali Riuniti di Foggia, University of Foggia, Via L Pinto, 71100, Foggia, Italy

## Abstract

A 21-year-old man presented with nasal obstruction of the right nasal fossa of 1 year duration. Nasal endoscopy revealed in the right inferior turbinate head a rounded neoplasm about 1 cm in diameter.

Cytologic study of a nasal scraping specimen disclosed numerous clusters containing columnar cells with cytomegaly, prominent multinucleation, markedly sparse shortened cilia; the cytoplasm contained an acidophil area and a small round area that stained poorly; cells with a large intracytoplasmic vacuole that was acidophil and PAS+. Serology tests using the nested polymer chain reaction (PCR) technique on serum, nasal and pharyngeal smears revealed an Epstein-Barr virus (EBV) infection that was confirmed at electron microscopy. The clinical and cytological features resolved 19 months after the initial evaluation.

**Conclusion:**

The authors advise carrying out clinical (endoscopy, serology, etc.) evaluation of all endonasal neoplasms and to routinely perform cytological study on nasal scraping specimens. When samples test positive for EBV, nasal and nasopharyngeal endoscopy should be performed regularly to detect possible evidence for nasopharyngeal carcinoma (NPC).

## Introduction

Introduced over a century ago, nasal cytology has become an indispensable diagnostic tool in the rhinology laboratory to differentiate various forms of rhino-pathologies, to follow the course of the disease and to monitor response to medical treatment [1-5].

In rhino-pathologies of viral origin, the microscopic picture is characterized by fairly aspecific cellular changes gathered under the term "ciliocytophthoria", which comprises degenerative alterations of the ciliary ultrastructure (shortening and focal or even general loss of the cilia), the cytoplasm (contraction of the cytoplasm, or even shortening of the upper portion of the cell body), the nucleus (chromatin margination with a ground-glass appearance and intranuclear inclusions) [6,7]. These cellular changes are usually accompanied by an equally aspecific infiltrate consisting chiefly of lymphocytes and neutrophils [8,9] and manifesting tissue inflammatory reaction.

The range of viruses that commonly infects the respiratory tract is notoriously wide (rhinovirus, coronavirus, respiratory syncytial virus [RSV], adenovirus, parainfluenza virus, coxsackievirus, cytomegalovirus). However, no specific cytomorphologic alteration been found to date that could represent a turning point in epidemiology, despite viral infections accounting for the bulk of human infectious diseases, or in prognosis and therapy. Some have strongly linked with the carcinogenesis of several tumor types, particularly Burkitt's lymphoma and nasopharyngeal carcinoma (NPC), or Epstein-Barr virus (EBV) [10-12].

The case described below focuses on specific microscopic and ultrastructural alterations in the nasal mucosal cells induced by EBV infection and draws on original findings.

## Case presentation

A 21-year-old man, student, non-smoker, came to our unit because of a nasal obstruction of the right nasal fossa of 1 year duration that was unaccompanied by other clinical symptoms (hyposomia, rhinorrhea, epistaxis) or signs pathognomic for allergy or rhinosinus inflammation.

**Figure 1:**
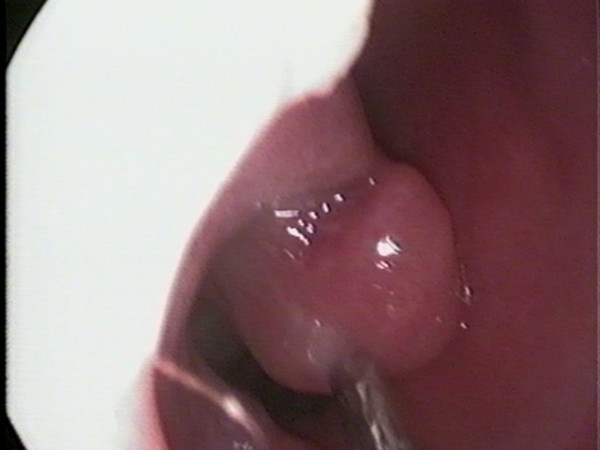
Nasal endoscopy: rounded neoplasm of inferior turbinate.

Nasal endoscopy revealed at the right inferior turbinate head a rounded neoplasm about 1 cm in diameter, pink in color, soft, not bleeding and not tender on palpation, covered with apparently healthy mucosa (Fig. 1). No other remarkable alterations in the other areas of the nasal cavity were found; the nasopharynx presented scars from a tonsillectomy performed when the patient was 7 years old.

Oropharyngeal, laryngoscopic and otoscopic evaluations were normal.

Active anterior rhinomanometry (150 Pascal) disclosed mildly elevated nasal resistance in both nasal sinuses; on decongestion testing with naphazoline values returned to normal in the left but not in the right nasal fossa (0.36 and 0.78, respectively).

The Prick test ruled out allergy toward common trophic and aeroallergens.

Cytologic studies of nasal scrapings obtained with the Rhino-probe^® ^were performed on specimens taken from the neoplasm and the mucosa of the inferior turbinate of both nasal cavities.

The cellular material was fixed in 95% ethyl alcohol for 4 minutes, and then stained using the May-Grünwald-Giemsa technique.

Slide observations were conducted at ×400 and ×1000 magnification.

**Figure 2:**
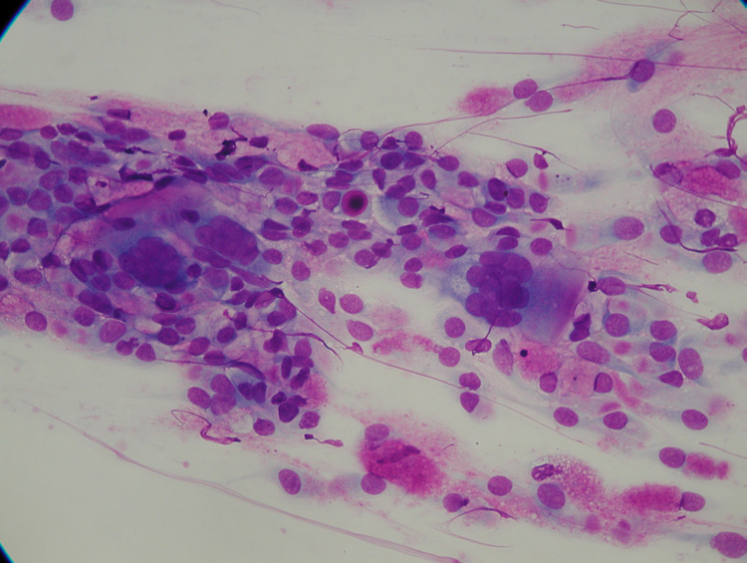
Numerous clusters containing columnar cells with cytomegaly and multinucleation. M.G.G. 400×.

**Figure 3:**
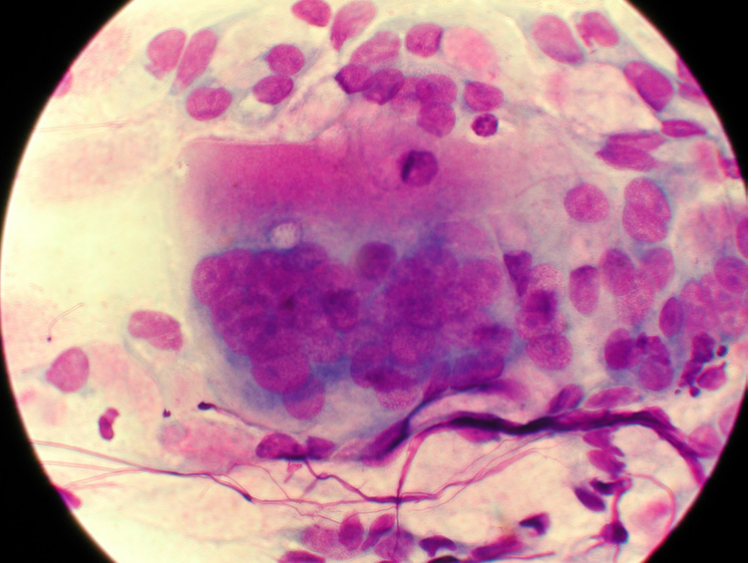
Columnar cell with citomegaly and multinucleation. M.G.G. 1000×.

Cytological determination disclosed a microscopic picture characterized by numerous clusters containing columnar cells with cytomegaly 5 to 6 times larger than normal (Fig. 2). The cellular elements were characterized by increased volume and pronounced multinucleation (12 nuclei were counted in some cells), vesicular chromatin with one or several nucleoli in the nucleus (Fig. 3).

The columnar multinuclear cells presented markedly a sparse and shortened ciliary ultrastructure.

Most of the multinuclear cells exhibited the following characteristics in the cytoplasm:

- an acidophil area of the apical region of the cytoplasm, with coarsely triangular morphology, with the apex oriented toward the nucleus.

- a small rounded weakly staining area.

**Figure 4:**
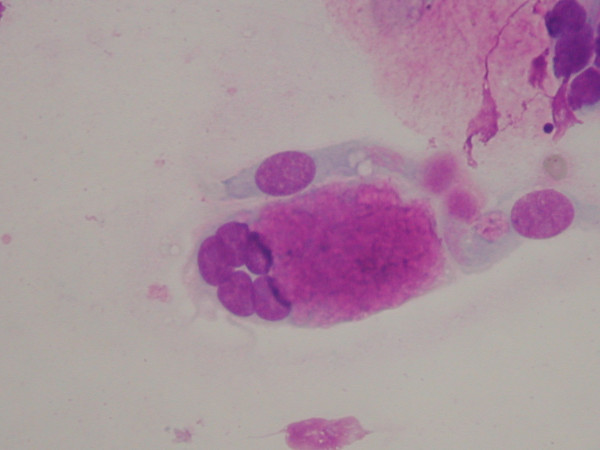
Muciparous globet cell with multinucleation. M.G.G. 1000×.

Multinucleation was also evident in the muciparous goblet cells, where nuclear chromatin was prominent due to the cytoplasma mucin pressing on the nuclei (Fig. 4).

**Figure 5:**
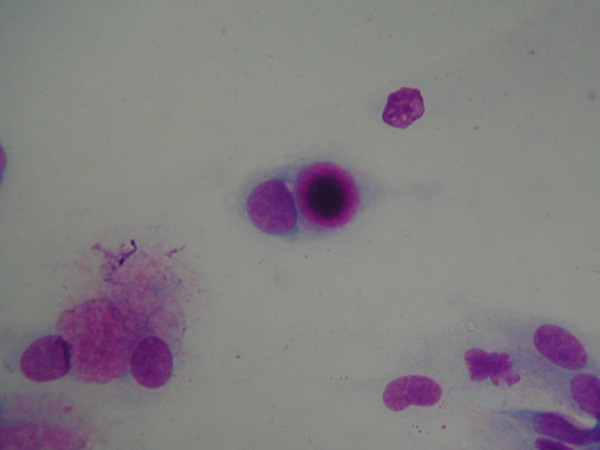
Columnar cells with a large acidophil intracytoplasmic vacuole. M.G.G. 400×.

Also present were columnar cells with a large acidophil intracytoplasmic vacuole. In some cells acidophilia was particularly intense in the center of the vacuole (Fig. 2, 5).

The vacuoles were positive for PAS staining.

Cellular alterations were found in all cytological specimens.

Based on the clinical and cytological findings, further serologic studies were performed to search for viral infection. Serologic tests were performed on blood serum and on nasal and pharyngeal smears using the nested polymerase chain reaction (PCR) technique to search for HHV6, VRS and EBV.

The serology detected an EBV infection, with viral presence on the nasal and pharyngeal smears and on the blood polymorphonucleates. Tests for HHV6 and VRS were negative.

The neoplasm was removed by endoscopy in local anesthesia. The histology report of the Institute of Anatomy and Histologic Pathology stated "fragment of nasal mucosa with pronounced angiectatic-edematous aspects of the stroma and inflammatory infiltration of the lymphoplasma cells and eosinophilia".

**Figure 6:**
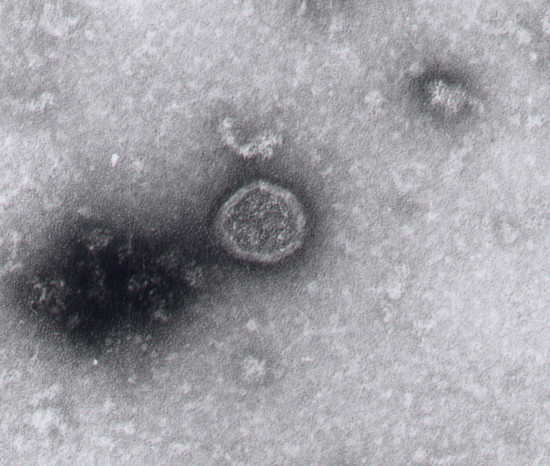
Electron Microscopy (128.000×): Epstein-Barr Virus inside multinuclear cells.

The ultrastructure study for the search for virus or viral particles conducted by the Electron Microscopy Center of the National Research Council, University of Bari, detected the presence of viral particles inside the cells of the nasal mucosa (Fig. 6).

At 3 months after initial examination, the patient returned for an outpatient control visit; nasal cytology monitoring and laboratory tests remained positive for EBV infection. At 19 months after the initial presentation, the infection finally cleared.

## Discussion

The respiratory tract is the principal route of access for most viral pathogens into the body. Several begin replicating in the nasal mucosa, sometimes without causing major clinical manifestations, but tending to produce systemic symptoms instead. Most viruses (rhinovirus, coronavirus, respiratory syncytial virus [RSV], adenovirus, parainfluenza virus) cause often benign respiratory illnesses, whereas others like EBV, coxsackie and cytomegalovirus produce much more severe diseases.

An important agent among the latter is the EBV which causes infectious mononucleosis (IM), which generally affects adolescents and young adults, and leads to severe pathologic syndromes such as lymphoproliferative syndrome, B cell lymphoma, Burkitt's lymphoma (BL), and nasopharyngeal carcinoma (NPC). Although NPC is relatively rare in Europe (1 case in 100,000 population) [11,13,14], the disease remains a diagnostic challenge because it is diagnosed late in the course of the disease, when the primary tumor has already manifested itself in secondary sites (laterocervical or retroangulomandibular metastasis) and/or loco-regional pathologies (recurrent tubotympanitis, chronic catarrhal otitis media, etc.) [15,17].

Our patient presented a clinically constant picture of vague symptoms consisting only of a mild but continuous monolateral nasal obstruction caused by a neoplasm involving the inferior turbinate. The site is highly unusual since endonasal neoplasms commonly affecting the middle turbinate or the ostio-meatal complex are nearly always benign (nasal polyps), secondary to vasomotor rhinopathies (NARES, nasal mastocytosis), and less often secondary to allergic or inflammatory rhinopathies (antro-coanal polyps). Only a very small percentage (3%) are malignant (inverted papilloma, leiomyosarcoma, nasopharyngeal carcinoma) [18,20].

In addition to the endoscopic aspects, what caught our interest were the cytological alterations characterized by multinucleation, which prompted us to conduct further studies. Cytologic inspection of the scraping specimen was the most specific method to investigate the cytopathology. Histologic determination was less specific in that it revealed only marked angiectasic-edematous phenomena of the stroma and eosinophil lymphoplasma cell inflammatory infiltration. That the finding was aspecific is obvious given the characteristics of the respiratory mucosa epithelium, which is composed of a pseudostratified pavimentous epithelium, with nuclear cells arranged at various heights; hence, epithelial cytomorphology does not permit the detection of multinucleation in histologic specimens. This aspect can be easily visualized by exfoliative cytology for the study of the specific morphology of each single cell.

Besides multinucleation, alterations in the cytoplasma were also found whose meaning we are unable to explain as regards the acidophil area in the apical portion of the multinucleate cells and the presence of cells with PAS+ vacuoles.

A particularly interesting finding uncovered by electron microscopy was the small rounded rarefied area inside the cytoplasma of several multinuclear ciliate columnar cells where the herpes virus concentration was highest.

These novel cellular alterations, described here for the first time, appear particular to EBV infection since they are absent in other viral infections of the nasal mucosa (adenovirus, rhinovirus, etc.) where we have consistently found (over 10,000 observations) only phenomena of "ciliocytophthoria", as mentioned above. The rare finding of EBV on the nasal mucosa corresponds to the equally low incidence of NPC in Western countries (1 case in 100,000 population).

Another important consideration is the clinical and prognostic aspect. It was interesting to find on repeated virological and cytological examinations of our patient a protracted persistence of EBV infection of the nasal mucosa, suggesting a chronic influenza on the cellular structures and surrounding connective tissues. This may provide important evidence for interpreting the proven evolution of viral infection toward the development of NPC [21,22]. Reports from the literature have, in fact, documented a strong link between NPC and EBV [10], and many types of dysplasia variously associated with concomitant tissue invasion often test EBV positive [23].

It has also been found that EBV is especially associated with less differentiated forms of NPC. PCR analysis of NPC biopsies have shown that EBV DNA is present in 100% of WHO type III (undifferentiated cells), but is less frequent in WHO type II (nonkeratinizing cells) and even less (20%) in WHO type I (keratinizing differentiated cells) [15,17].

While EBV has been occasionally identified in the epithelium adjacent to invasive tumors, which sometimes exhibits apparently normal, hyperplastic or metaplastic features, it has never been found in biopsies of histological nasopharyngeal specimens from patients without NPC [11].

Preinvasive lesions have shown to test positive for clonal EBV DNA, thus supporting the hypothesis that EBV infection is very early and probably initiates the development of NPC. In light of these findings we can say that nasopharyngeal biopsies for EBV screening may be a useful aid in the early diagnosis of NPC [10,11].

An intriguing element in our case was the proliferative aspect of the nasal mucosa stimulated by the virus, with the presence of hyperplastic tissue confined to the inferior turbinate. This suggests extreme caution in the diagnosis of nasopharyngeal neoplasms especially in adults. In the hypothesis of an EBV viral pathogenesis of a neoplasm, examination of the biopsy material should not be limited exclusively to histological study to rule out NPC.

In cases where its presence is not confirmed, it is wise to conduct cytological studies on several samples of the neoplasm and the surrounding tissues to confirm the alterations described above that may be pathologically significant for EBV infection. Findings of this type call for close monitoring of the patient and follow-up cytological studies that will check for the persistence of viral infection and detect the onset of malignant transformation of tissues affected by an EBV infection. Early diagnosis offers optimum chances for prompt treatment, considering the high sensitivity of NPC to radiation therapy of the localized forms of the cancer.

In conclusion we feel that in order to confirm the correlation between our clinical and cytological findings and nasopharyngeal cancers, mass screening programs and clinical follow up will be necessary, particularly in those areas of the world (southern China and Southeast Asia) where these diseases have a higher incidence (20 to 30 cases in 100,000 population).
